# Case Report: Octreotide plus CVD chemotherapy for the treatment of multiple metastatic paragangliomas after double resection for functional bladder paraganglioma and urothelial papilloma

**DOI:** 10.3389/fonc.2022.1072361

**Published:** 2023-01-20

**Authors:** Zilong Wang, Feifan Liu, Chao Li, Huisheng Yuan, Yuzhu Xiang, Chunxiao Wei, Dongyuan Zhu, Muwen Wang

**Affiliations:** ^1^ Department of Andrology, The Seventh Affiliated Hospital, Sun Yat-sen University, Shenzhen, China; ^2^ Department of Urology, Shandong Provincial Hospital, Cheeloo College of Medicine, Shandong University, Jinan, China; ^3^ Cancer Center, Shandong Cancer Hospital and Institute, Shandong First Medical University and Shandong Academy of Medical Sciences, Jinan, China; ^4^ Department of Urology, Shandong Provincial Hospital Affiliated to Shandong First Medical University, Jinan, China; ^5^ Rare Tumors Department, Shandong Cancer Hospital and Institute, Shandong First Medical University and Shandong Academy of Medical Sciences, Jinan, China

**Keywords:** metastatic paraganglioma, octreotide, chemotherapy, PET/CT, SSTR2, case report

## Abstract

**Background:**

Metastatic pheochromocytomas and paragangliomas are rare neuroendocrine tumors with a poor prognosis. Bladder paraganglioma concomitant with urothelial papilloma is even rarer. However, the rate of tumor response to cyclophosphamide–vincristine–dacarbazine (CVD) chemotherapy and 5-year overall survival for patients with metastatic PPGLs remained lower. We described, for the first time, a case of a patient with multiple metastatic bladder PGL who received octreotide LAR combined with CVD chemotherapy after urological surgery and then octreotide therapy was continued during follow-up.

**Case presentation:**

A 43-year-old male patient was admitted to the urology department for frequent micturition syncope concomitant with malignant hypertension. Preoperative findings were elevated levels of normetanephrine in 24-h urine or plasma. CT and MRI indicated diagnosis of suspicious bladder paraganglioma. Transurethral resection of bladder tumor combined with laparoscopic partial cystectomy was performed successfully after preoperative phenoxybenzamine with aggressive volume repletion for 7 days. The result of postoperative pathology was immediate-risk functional bladder paraganglioma (T2N0M0, Stage II) concomitant with urothelial papilloma, and the immunohistochemistry results of PPGL were positive for Ki-67 (15%), SDHB, CgA, and SSTR2. The patient achieved enhanced recovery with normal urination and no syncope after surgery. However, the results of ^18^F-FDG and ^18^F-DOTATATE PET/CT found that the metastatic localizations of bladder PGLs were in the liver, lung, and bones at the 8th month after surgery. The patient received octreotide long-acting repeatable plus six courses of CVD chemotherapy for 6 months, and then octreotide therapy was continued every 3 months until now. Metastatic localizations were stable in CT scans, and vanillylmandelic acid in 24-h urine was maintained at lower levels during follow-up.

**Conclusion:**

Octreotide long-acting repeatable plus CVD chemotherapy after surgery could achieve stable disease in the case with multiple metastatic bladder PGLs, and the following octreotide therapy could maintain a state of stable disease during the period of 6-month follow-up.

## 1 Introduction

Pheochromocytomas and paragangliomas (PPGLs) of the bladder are among the rarest types of thoracoabdominal neuroendocrine tumors (NETs), originating from the neural crest within the bladder wall (1), accounting for approximately 0.7% of all pathological types of PPGLs and less than 0.05% of all bladder tumors (2, 3). Bladder paraganglioma (PGL) concomitant with urothelial papilloma is even rarer. However, 14% of patients with bladder PGLs experienced recurrence and metastasis after partial cystectomy or transurethral resection of bladder tumor (TURBT) (4). In the presence of metastatic PPGLs, the 5-year overall survival (OS) rate was 50%. Cytoreductive resection, including cyclophosphamide–vincristine–dacarbazine (CVD) chemotherapy, was recommended as the mainstay of treatment for unresectable and metastatic PPGLs (5). However, approximately 33% of patients with metastatic PPGLs exhibited a tumor response to CVD chemotherapy (5). The OS rate for responders at 5 years was 51% (6) and the median response duration was 1.3 years (7). Therefore, future in-depth studies on postoperative adjuvant therapeutic strategies for multiple metastatic PPGL are needed to maintain a state of stable disease and further improve prognosis after CVD chemotherapy.

Octreotide is a somatostatin analog (SSA) that suppresses the proliferation of NETs through specifically binding to somatostatin receptors (SSTRs) and alleviates the clinical manifestations of metastatic PPGLs (8). Octreotide long-acting repeatable (LAR) has been recommended to potentially control tumor growth in patients with metastatic neuroendocrine midgut tumors based on the PROMID studies (9, 10). Meanwhile, the radiographic agents of somatostatin receptors, including ^68^Ga/^18^F/^177^Lu-DOTA-D-Phe1-Tyr3-octreotate (DOTATATE) and ^18^F-AlF-1,4,7-triazacyclononane-1,4,7-triacetic acid-octreotide (^18^F-AlF-NOTA-octreotide), have diagnostic and treatment significances for metastatic lesions in NETs (11–16). Therefore, the National Comprehensive Cancer Network (NCCN) guidelines recommended peptide receptor radionuclide therapy (PRRT) with ^177^Lu-DOTATATE or octreotide therapy as a treatment option for patients with PPGLs that are SSTR-positive upon imaging (5). However, PPRTs have not been widely applied as a result of high technically demanding and heavy economic burden.

Nowadays, octreotide LAR has been approved by the Food and Drug Administration (FDA) for unresectable and metastatic gastroenteropancreatic neuroendocrine tumors (GEP-NETs) that are SSTR-positive with imaging (17). Patients with hormonally functional NETs should continue octreotide along with PRRT or octreotide LAR (5). However, whether octreotide therapy combined with CVD chemotherapy could achieve better efficacy in patients with hormonally functional, unresectable, and multiple metastatic bladder PGL that is SSTR-positive upon imaging has not been reported. We describe, for the first time, a case of a patient with multiple metastatic bladder PGL who received octreotide LAR combined with CVD chemotherapy after double resection, and then continued octreotide therapy during follow-up.

## 2 Case description

### 2.1 Preoperative condition

**Figure 1:**
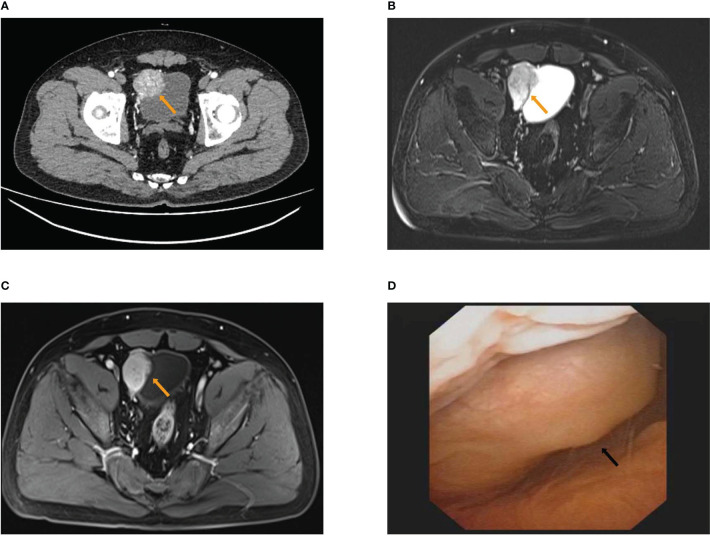
The results of preoperative examination. **(A)** The computed tomography urography (CTU) scan showed a roundish soft tissue lesion (4.8 cm × 3.7 cm) in the right anterior wall of bladder with heterogeneous enhancement of arterial phase. **(B, C)** MRI showed an approximately circular signal of high T2WI (4.9 cm × 3.8 cm) in the right anterior wall of bladder **(B)** with heterogeneous enhancement of arterial phase **(C)**. **(D)** Cystoscopy showed inward right anterior wall.

A 43-year-old male patient was admitted to the Department of Urology with frequent micturition syncope concomitant with hypertension for several weeks on 4 November 2020. The patient was free of diabetes mellitus and other chronic cerebrovascular disorders. The patient also had no history of psychological, genetic, or other disorders and no family history of malignant neoplasms. The patient was taking irbesartan and amlodipine for hypertension and angina pectoris for more than 1 year. The physical examination was normal. Clinical laboratory tests including blood routine examination, blood sugar, blood lipid, serum electrolytes, liver function, and renal function were also normal. Routine urine examination of red blood cells was 5.5/HPF (normal range ≤3/HPF). Serum endocrine biomarkers were as follows: normetanephrine (NMN) 18,063.8 pmol/L (normal range ≤709.7 pmol/L), metanephrine (MN) 194.9 pmol/L (normal range ≤420.9 pmol/L), norepinephrine (NE) 8,590.5 pmol/L (normal range 413.9–4,434.2 pmol/L), epinephrine (E) 151.8 pmol/L (normal range ≤605.9 pmol/L), aldosterone (ALD) 232.19 pg/ml (normal range 40–310 pg/ml), and cortisol (COR) 347.00 nmol/L (normal range 160–660 nmol/L). Urine endocrine biomarkers were as follows: NMN in 24-h urine 5,630 nmol/24 h (normal range <312 nmol/24 h) and MN in 24-h urine 79 nmol/24 h (normal range <216 nmol/24 h). The computed tomography urography (CTU) scan indicated a bladder tumor with a sub-circular soft tissue lesion (4.8 cm × 3.7 cm) in the right anterior wall of the bladder with heterogeneous enhancement in the arterial phase and decreased enhancement of venous phase and excretory phase (**Figure 1A**; **Supplementary Figures 1A–C**). Magnetic resonance imaging (MRI) also indicated bladder tumor with an approximately circular signal of high T2-weighted imaging (T2WI) (4.9 cm × 3.8 cm) in the right anterior wall of the bladder (**Figures 1B** **, C**; **Supplementary Figures 1D–F**). Cystoscopic findings revealed a cauliflower-shaped mass with the right anterior wall of the bladder directed inward and a tip in the right wall, approximately 0.8 cm in diameter (**Figure 1D**; **Supplementary Figure 1G**). The preoperative diagnosis was suspicious bladder PGL concomitant with bladder tumor.

### 2.2 Operative condition and pathologic results

**Figure 2:**
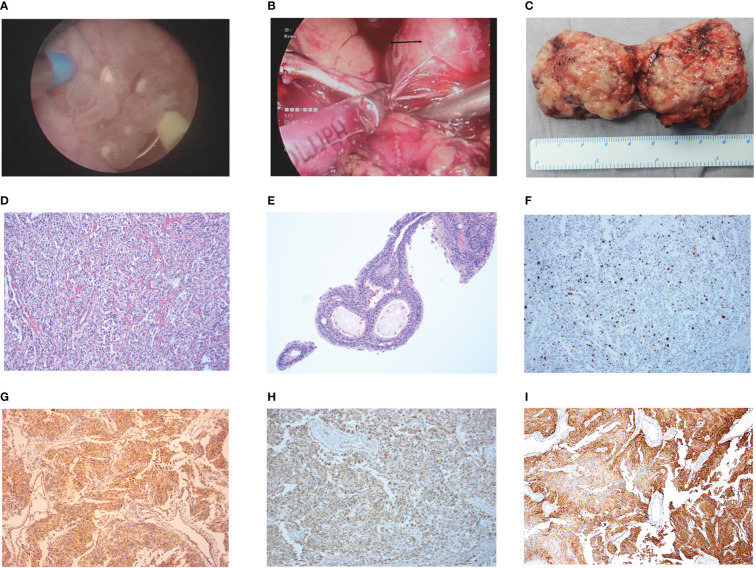
The intraoperative condition and postoperative pathology results. **(A)** Intraoperative condition of urothelial papilloma in TURBT. **(B)** Intraoperative condition of bladder pheochromocytomas in laparoscopy-assisted partial cystectomy. **(C)** The postoperative pathology tissues of bladder pheochromocytomas. **(D, E)** The results of postoperative pathology with HE staining in bladder pheochromocytomas **(D)** and urothelial papilloma **(E)**. **(F–I)** The results in IHC staining of Ki-67+ (15%) **(F)**, SDHB(+) **(G)**, CgA(+) **(H)**, and SSTR2(+) **(I)** in bladder pheochromocytomas.

The patient received preoperative phenoxybenzamine with aggressive volume repletion for 7 days. The patient successfully underwent laparoscopic partial cystectomy combined with TURBT (**Figures 2A** **–C**). The postoperative pathology report confirmed bladder PGL (T2N0M0, Stage II) concomitant with urothelial papilloma (**Figures 2D** **, E**), with immunohistochemistry (IHC) positive for Ki-67 (15%), chromogranin A (CgA), succinate dehydrogenase B (SDHB), somatostatin receptor 2 (SSTR2), and synapsin (Syn) of bladder PGL as well as CK20 (umbrella cells) and Ki-67 (1%) of urothelial papilloma (GAPP score of 6), and negative for S-100 of bladder PGL (**Figures 2F–I**; **Supplementary Figures 2A–D**). The final diagnosis was intermediate-risk functional bladder PGL concomitant with urothelial papilloma. The patient achieved enhanced recovery after surgery and returned to normal clinical manifestations, including normal urination and no syncope.

### 2.3 Postoperative recurrence condition

**Figure 3:**
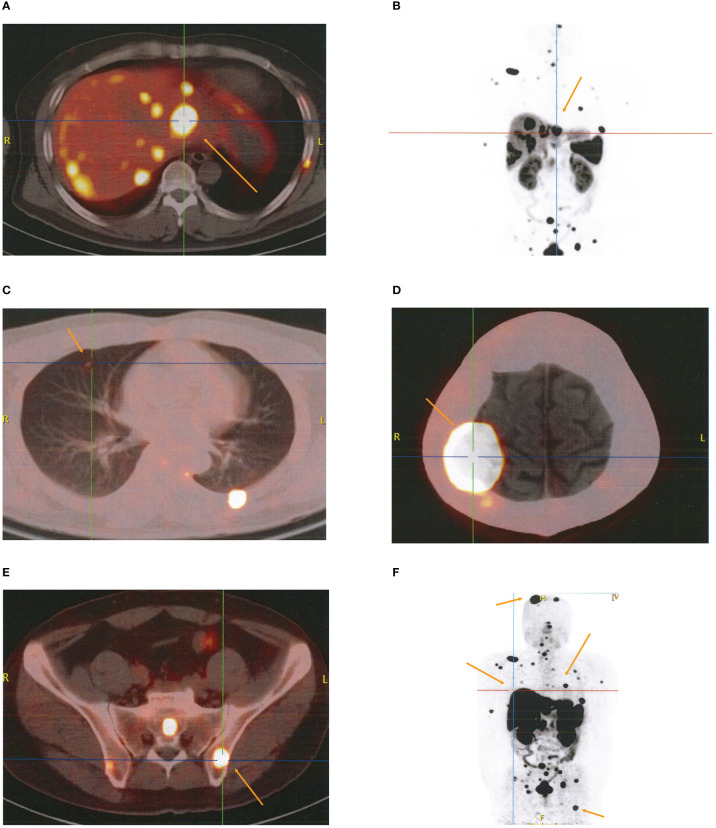
The results of ^18^F-DOTATATE PET/CT for metastatic localizations of paragangliomas. **(A–C)** Multiple high uptake localizations in liver **(A, B)** and lung **(C)**. **(D, E)** High uptake localizations with osteolytic bone destruction of right parietal skull bone **(D)** and ilium **(E)**. **(F)** Whole-body SPECT/CT imaging.

**Figure 4:**
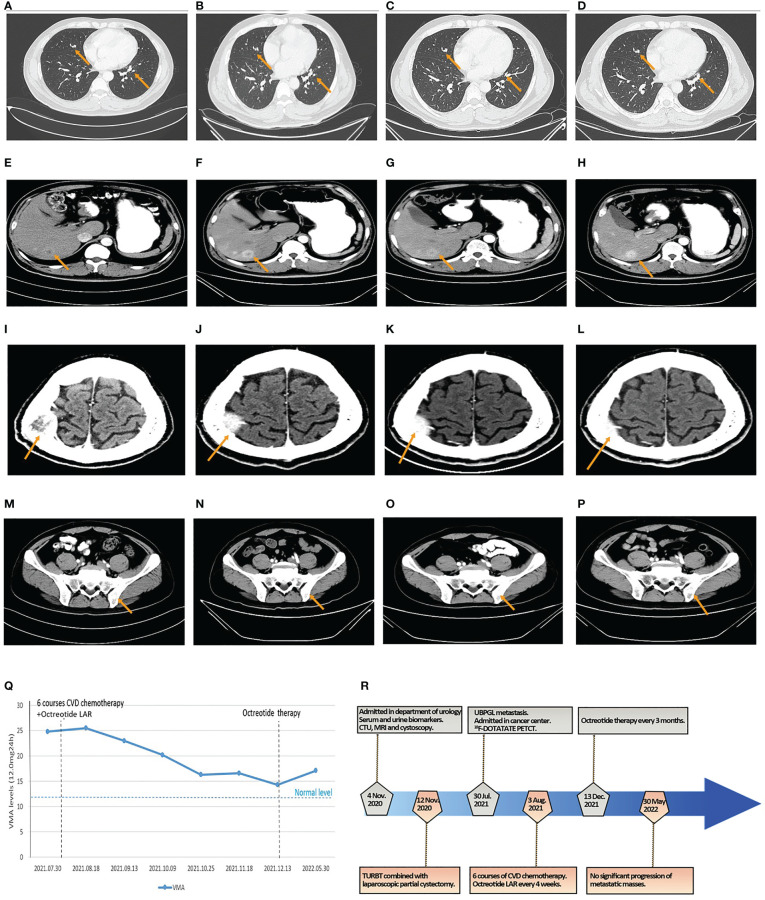
The results of CT and VMA in 24-h urine during octreotide plus six courses of CVD chemotherapy and the flowchart of the timeline for diagnosis and treatment process. **(A–D)** The size of metastatic localizations of lung had no significant progression on two courses **(A)**, four courses **(B)**, and six courses of chemotherapy **(C)**, and 6-month follow-up **(D)**. **(E–H)** The size of metastatic localizations of liver had no significant progression on two courses **(E)**, four courses **(F)**, and six courses of chemotherapy **(G)**, and 6-month follow-up **(H)**. **(I–L)** The size of metastatic localizations of skull had no significant progression on two courses **(I)**, four courses **(J)**, and six courses of chemotherapy **(K)**, and 6-month follow-up **(L)**. **(M–P)** The size of metastatic localizations of illum had no significant progression on two courses **(M)**, four courses **(N)**, and six courses of chemotherapy **(O)**, and 6-month follow-up **(P)**. **(Q)** The line graph of VMA levels in 24-h urine changes in the perioperative and chemotherapeutic period. **(R)** The diagnosis and treatment flowchart.

The patient was admitted to the Cancer Center because a skull mass was found on the 8th month after surgery without regular follow-up. The ^18^F-FDG positron emission tomography (PET)/CT findings indicated multiple high uptake liver (2.8 cm × 2.4 cm) with a maximal standardized uptake value (SUVmax) of 17.7 and bilateral pulmonary nodules with a maximum diameter of 0.5 cm (**Supplementary Figures 3A–C**), as well as high uptake masses and osteolytic bone destruction in the right parietal skull bone, vertebral body, and ilium (**Supplementary Figures 3D, E**). These results revealed that the metastatic localizations of bladder PGLs were in the liver, lung, and bones. The patient underwent ^18^F-DOTATATE PET/CT targeted imaging as a result of the positive expression of SSTR2. The results showed multiple high uptakes of liver and lung nodules with a SUVmax of 65.0 and 2.2, respectively (**Figures 3A** **–C**), as well as a high uptake mass with osteolytic bone destruction of the right parietal skull and iliac bone (**Figures 3D** **–F**). These results of ^18^F-DOTATATE PET/CT confirmed the same diagnosis. The level of vanillylmandelic acid (VMA) in 24-h urine was 24.80 mg/24 h (normal range ≤12.00 mg/24 h) (**Figure 4Q**).

### 2.4 Treatment strategy and efficacy

The patient received six courses of CVD chemotherapy with cyclophosphamide (1.4 g day 1), vincristine (2 mg day 1), and dacarbazine (0.4 g days 1–5) on 3 August 2021, every 16–26 days (mean 22 days). Considering the positive expression of SSTR2 in PGL tissues and the high uptake of octreotide on ^18^F-DOTATATE PET/CT in multiple metastases, the patient was simultaneously subjected to octreotide LAR (30 mg intramuscularly every 4 weeks) based on the recommendation of NCCN guidelines and the PROMID studies (5, 9, 10). The results of CT scans showed no significant progression in the size of the lung, liver, skull, and ilium metastases (**Figures 4A** **–L**). The levels of VMA in 24-h urine slowly declined during this period (**Figure 4Q**). Therefore, octreotide LAR plus CVD chemotherapy could achieve stable disease of multiple metastatic bladder PGL.

After finishing the sixth course of CVD chemotherapy and the latest treatment of octreotide LAR on 13 December 2021, the patient continued octreotide therapy (30 mg intramuscularly every 3 months) until now to control hormonally functional PGL. CT scans showed no significant progression of metastatic masses in the lung, liver, skull, and ilium (**Figures 4M** **–P**), and the level of VMA in 24-h urine was 17.10 mg/24 h after 6 months (**Figure 4Q**). The patient maintained a state of stable disease during the period of 6-month follow-up. The flowchart of timeline for diagnosis and the treatment process is shown in **Figure 4R**.

## 3 Discussion

We described, for the first time, a case of a patient with metastatic bladder PGL who achieved stable disease after octreotide plus six courses of CVD chemotherapy. The patient received laparoscopic partial cystectomy and TURBT after preoperative phenoxybenzamine with volume repletion for bladder tumors. Postoperative diagnosis was the immediate-risk functional bladder PGL concomitant with urothelial papilloma. On the 8th month after surgery, ^18^F-DOTATATE PET/CT showed that the metastatic localizations of bladder PGLs were in the liver, lung, and bones. The patient received octreotide LAR every 4 weeks combined with six courses of CVD chemotherapy for 6 months and then octreotide therapy was continued every 3 months. The patient achieved and maintained a state of stable disease during the 6-month follow-up.

Bladder PGLs refer to the ectopic pheochromocytomas (PCCs) that arise from sympathetic ganglia of the bladder (18). Approximately 40% of functional PGLs secrete catecholamines (CA) and their metabolites metanephrine (MN) and normetanephrine (NMN) (5), resulting in “quadruple signs”, headache and/or micturition syncope, cold sweating, palpitation and/or persistent hypertension, and hematuria (19). Elevated levels of MN or NMN are suggestive of PCCs or PGLs, and elevations in MN or NMN levels that are three times above the upper limit of normal are diagnostic (5). In general, adrenal PCCs more commonly secrete MN and PGLs secrete NMN (18). In the present case report, the results of 24-h urine or plasma showed that there were elevated levels of NMN and normal levels of MN. Therefore, the patient was classified as functional bladder PGL in combination with the typical hormonal symptoms.

Surgical resection is the mainstay of treatment for bladder PGLs, and surgery can cause a sudden release of large amounts of catecholamines, causing very significant and sometimes life-threatening hypertension (5). However, the prognosis and metastasis rates of bladder PGLs are not affected by the initial surgical choice (20). TURBT can trigger intraoperative hypertensive crisis due to the stimulus of sympathetic plexus, which is distributed in all layers of the bladder walls. Thus, some surgeons choose partial or radical cystectomy as the mainstay of treatment for bladder PGLs. However, adequate preoperative patient preparation ensured the feasibility and safety of TURBT in patients with bladder PGLs (21). In the present case report, radical cystectomy would have increased surgical trauma to increase the risk of lower quality of life. As for the different locations of uroepithelial papilloma with a maximum size of 0.8 cm, the patient received a laparoscopic partial cystectomy combined with TURBT after receiving phenoxybenzamine with aggressive volume repletion for 7 days, and his intraoperative vital signs were stable.

Surveillance for patients with bladder PGLs should be strictly standardized to monitor tumor recurrence and metastasis. There is a lack of data to clearly state the postoperative recurrence and metastasis rates of bladder PGLs. A systematic review by Beilan et al. and a multicenter study of 110 patients by Yu et al. found a metastasis rate of 14.2% and 30% for bladder PGLs, respectively (4, 20). In this case report, the patient showed the occurrence of distant multiple metastasis in bones and visceral organs on the 8th month after the surgical strategy due to irregular surveillance. Therefore, a strict standard surveillance should be developed for these patients with immediate or high-risk functional bladder PGLs. Following complete resection, complete patient history and physical (H&P) examination should be performed and blood pressure and tumor markers (NMN or MN in 24-h urine or plasma) should be measured after 12 weeks to 12 months and then every 6 to 12 months for the first 3 years, and then annually for up to 10 years. After 10 years, surveillance should be considered as clinically indicated (5). For locally unresectable disease or distant metastases of bladder PGLs, chest/abdominal/pelvic CT scans with contrast should be monitored every 3 months for the first year, then every 6 months for the next year, and then annually up to 5 years (4). In addition, FDG-PET/CT scans or SSTR-based imaging can be considered (5). Meanwhile, Katiyar et al. also recommended that all patients with bladder PGLs should have regular cystoscope examinations (22).

Urine or plasma catecholamines are no longer routinely recommended for the evaluation of PGLs as 15% to 20% of patients with PGLs have normal levels of urine catecholamines due to intermittent secretion in some tumors and insignificant secretion by others (5). VMA is the primary end metabolite of catecholamines, so its measurement in 24-h urine has long been used for diagnosis and surveillance of PGLs. The relative rise of VMA levels in the presence of PGLs is much less dramatic than the rise seen in the levels of NMN or MN, and the sensitivity of urine VMA levels is therefore low (below 65%). However, the specificity of the test is high, especially in nonfamilial cases (99%) (23). Therapy with various modalities produces a reduction in catecholamine metabolite excretion in most patients (23). In the present case report, the patient received octreotide LAR plus CVD chemotherapy and the following octreotide therapy in the Cancer Center. Unfortunately, the patient was only monitored by 24-h urine VMA as an alternative marker for lack of requisite equipment and technology. The continuous decline of 24-h VMA also indicated the positive therapeutic effects of multiple metastatic bladder PGLs along with the results of CT scans.

Pathological markers and the timing of metastasis development after initial diagnosis are essential factors affecting the prognosis of patients with metastatic bladder PGLs. High Ki-67 proliferation index is associated with more aggressive clinical courses and worse prognosis (24–26). Although the World Health Organization (WHO) defines a Ki-67 index of 3%–20% as intermediate-grade (G2) PPGLs, this index did not accurately describe prognosis. Nuñez-Valdovinos et al. recommended the Ki-67 index exceeding 10% as G2 PPGLs (27). In this case report, the patient experienced multiple metastases on the 8th month after surgery, although he was diagnosed as G2 bladder PGLs with a Ki-67 index of 15%. According to the study of Hamidi et al. (28), the timing of metastatic development after the initial diagnosis played an important role in the prognosis of metastatic bladder PGLs. Patients who had synchronous metastases had a 7%–48% of metastasis rate and a 3.7-year OS, while the median OS of metachronous metastases was 9.9 years (6, 20). Metastatic PGL was found on the patient on the 8th month after surgery, and it was classified as metachronous metastasis.

Because bladder PGL is insensitive to both chemotherapy and radiotherapy, CVD chemotherapy is used in metastatic bladder PGLs as a palliative chemotherapeutic procedure to delay disease progression, but the rate of tumor response to CVD chemotherapy is 33% (5) and the effective rate is only 37% (29). ^131^I-MIBG and ^68^Ga-DOTATATE have been approved by the FDA as radiopharmaceuticals for diagnostic imaging and targeted therapy, with higher sensitivity and specificity, respectively (30, 31). However, less than 40% of patients responded to the modality of ^131^I-MIBG (32) and several types of octreotide PET/CT had a higher sensitivity of malignant and metastatic lesions than ^131^I-MIBG. The radionuclides ^18^F and ^177^Lu showed the same effects as ^68^Ga on octreotide radiopharmaceuticals (13–16, 33). In this case report, the sensitivity and specificity of ^18^F-DOTATATE PET/CT were higher than those of ^18^F-FDG PET/CT. Therefore, ^18^F-DOTATATE PET/CT has a high diagnostic value for this case with metastatic PGLs. However, these strategies of targeted radiotherapy, including ^177^Lu-DOTATATE, ^131^I-MIBG, and ^68^Ga-DOTATATE, have not been widely applied as a result of high technically demanding and heavy economic burden.

Octreotide, as a somatostatin analog (SSA), was applied in nuclear imaging and inhibition of proliferation and clinical manifestations of PPGLs through specifically binding to somatostatin receptor 2 (SSTR2). The expression of SSTR2 was positive in more than 93% of metastatic PGLs (34, 35), and SSTR2 mediates both the biochemical and antiproliferative effect of octreotide (36). Octreotide exerts anti-tumor effects *via* binding to SSTR2; upregulating TRAIL, DR4 and TNFRI; downregulating Bcl-2; promoting cell apoptosis; inhibiting cytokine release, CA, and other hormone synthesis; and tumor angiogenesis (37). Meanwhile, octreotide could promote the mitogenic effect of vincristine. This case report showed that the patient had multiple metastatic bladder PGLs positive for SSTR2, which was confirmed through high uptake of ^18^F-DOTATATE PET/CT. The NCCN guidelines recommended PRRT with^177^Lu-DOTATATE or octreotide therapy as a treatment option for patients with PPGLs that are SSTR-positive upon imaging, and patients with hormonally functional NETs should continue octreotide along with PRRT or octreotide LAR (5). Thus, we performed six courses of CVD chemotherapy plus octreotide LAR for 6 months and then continued octreotide therapy for the next 6 months as a result of the technical limitation of ^177^Lu-DOTATATE. The patient achieved and maintained a state of stable disease during the period of six courses of CVD chemotherapy and 1-year octreotide therapy. Meanwhile, it is important to perform octreotide PET/CT and SSTR2 IHC staining as predictive tests before initiation of treatment.

This study has the following limitations: Firstly, the time of follow-up is not enough to evaluate the efficacy of CVD chemotherapy plus octreotide in the prognosis of multiple metastatic bladder PGL. We only draw a conclusion that this treatment strategy can achieve stable disease. This patient should require longer follow-up in maintaining a state of stable disease. Secondly, due to the lack of requisite equipment and technology, we did not detect NMN in 24-h urine or plasma for this patient when he was found to have multiple metastatic PGLs. The patient was only monitored by 24-h urine VMA as an alternative marker. Thirdly, the patient did not receive gene testing in the perioperative period. Considering that the patient had a functional bladder PGL concomitant with urothelial papilloma, our team suggested that the patient receive gene testing. However, the patient refused our suggestion. SDHB gene mutations are associated with a 40% to 60% risk of developing metastatic PGLs (20, 28, 38–40). Although the patient showed no mutation of SDHB, we also recommended genetic testing in patients with bladder PGLs to better guide adjuvant therapy and to exclude family inheritance. Finally, the metastatic tissues were also not taken for further molecular biological analyses. Further studies are needed to investigate the specific mechanism of octreotide in the treatment of metastatic bladder PGLs in the future.

## 4 Conclusion

Octreotide LAR plus CVD chemotherapy after surgery could achieve stable disease in the case with multiple metastatic bladder PGLs, and the following octreotide therapy could maintain a state of stable disease during the period of 6-month follow-up.

## Data availability statement

The original contributions presented in the study are included in the article/**Supplementary Material**. Further inquiries can be directed to the corresponding authors.

## Ethics statement

Written informed consent was obtained from the individual(s) for the publication of any potentially identifiable images or data included in this article.

## Author contributions

ZW and FL provided patient information and wrote the manuscript. FL, CL, HY, and CW collected the data. ZW, HY, and CW prepared histopathological examination and illustrations. CL, YX, DZ, and MW consulted the treatment plan. DZ and MW reviewed the topic presentation, structure of the manuscript, illustrations, and photographs. DZ and MW obtained resources and reviewed and edited the writing. All authors contributed to the article and approved the submitted version.

## Supplementary material

Supplementary Figure 1 The results of preoperative examination. **(A–C)** The Computed Tomography Urography (CTU) scan showed roundish soft tissue lesion (4.8cm×3.7cm) in the right-anterior wall of bladder with decreased enhancement of venous phase and excretory phase. **(D–F)** MRI showed an approximately circular signal of high T2WI (4.9cm×3.8cm) in the right-anterior wall of bladder with decreased enhancement of excretory phase. **(G)** Cystoscopy showed pedunculated, cauliflower-like mass in right lateral wall of bladder with diameter of about 0.8cm.

Supplementary Figure 2 The intraoperative condition and postoperative pathology results. **(A, B)** The results of postoperative pathology with Syn(+) **(A)** and S-100 (–) **(B)** in IHC staining of bladder pheochromocytomas. **(C, D)** The results of postoperative pathology with HE staining and CK20+(umbrella cells) **(C)** and Ki-67+(1%) **(D)** in IHC staining of urothelial papilloma.

Supplementary Figure 3 The results of ^18^F-FDG PET/CT for metastatic localizations of paragangliomas. **(A–C)** Multiple high uptake liver **(A, B)** and lung nodule **(C)**. **(D, E)** High uptake localizations with osteolytic bone destruction of right parietal skull bone **(D)**, vertebral body and ilium **(E)**.
