# Supplementary material for: Case Report: Octreotide plus CVD chemotherapy for the treatment of multiple metastatic paragangliomas after double resection for functional bladder paraganglioma and urothelial papilloma
Source: Front Oncol. 2023 Jan 20;12:1072361. doi: 10.3389/fonc.2022.1072361 (PMC9895770; doi:10.3389/fonc.2022.1072361)
Supplement: Supplementary Figure 1 — The results of preoperative examination. (A–C) The Computed Tomography Urography (CTU) scan showed roundish soft tissue lesion (4.8cm×3.7cm) in the right-anterior wall of bladder with decreased enhancement of venous phase and excretory phase. (D–F) MRI showed an approximately circular signal of high T2WI (4.9cm×3.8cm) in the right-anterior wall of bladder with decreased enhancement of excretory phase. (G) Cystoscopy showed pedunculated, cauliflower-like mass in right lateral wall of bladder with diameter of about 0.8cm. [file Image_1.pdf]

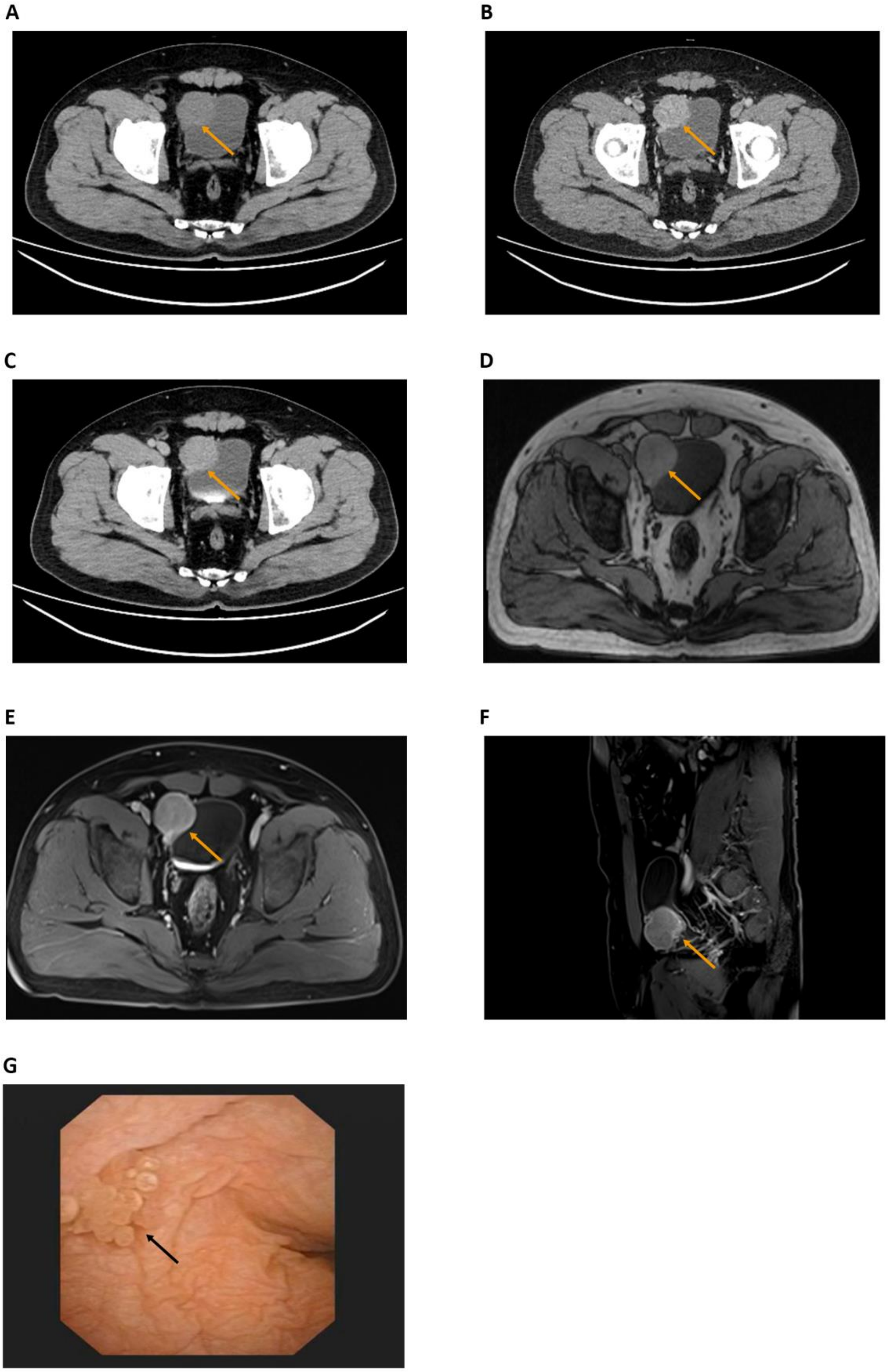

SUPPLEMENTARY FIGURE 1 The results of preoperative examination. (A–C) The Computed Tomography Urography (CTU) scan showed roundish soft tissue lesion (4.8cm×3.7cm) in the right-anterior wall of bladder with decreased enhancement of venous phase and excretory phase. (D–F) MRI showed an approximately circular signal of high T2WI (4.9cm×3.8cm) in the right-anterior wall of bladder with decreased enhancement of excretory phase. (G) Cystoscopy showed pedunculated, cauliflower-like mass in right lateral wall of bladder with diameter of about 0.8cm.
