# Supplementary material for: Case Report: Octreotide plus CVD chemotherapy for the treatment of multiple metastatic paragangliomas after double resection for functional bladder paraganglioma and urothelial papilloma
Source: Front Oncol. 2023 Jan 20;12:1072361. doi: 10.3389/fonc.2022.1072361 (PMC9895770; doi:10.3389/fonc.2022.1072361)
Supplement: Supplementary Figure 2 — The intraoperative condition and postoperative pathology results. (A, B) The results of postoperative pathology with Syn(+) (A) and S-100 (–) (B) in IHC staining of bladder pheochromocytomas. (C, D) The results of postoperative pathology with HE staining and CK20+(umbrella cells) (C) and Ki-67+(1%) (D) in IHC staining of urothelial papilloma. [file Image_2.pdf]

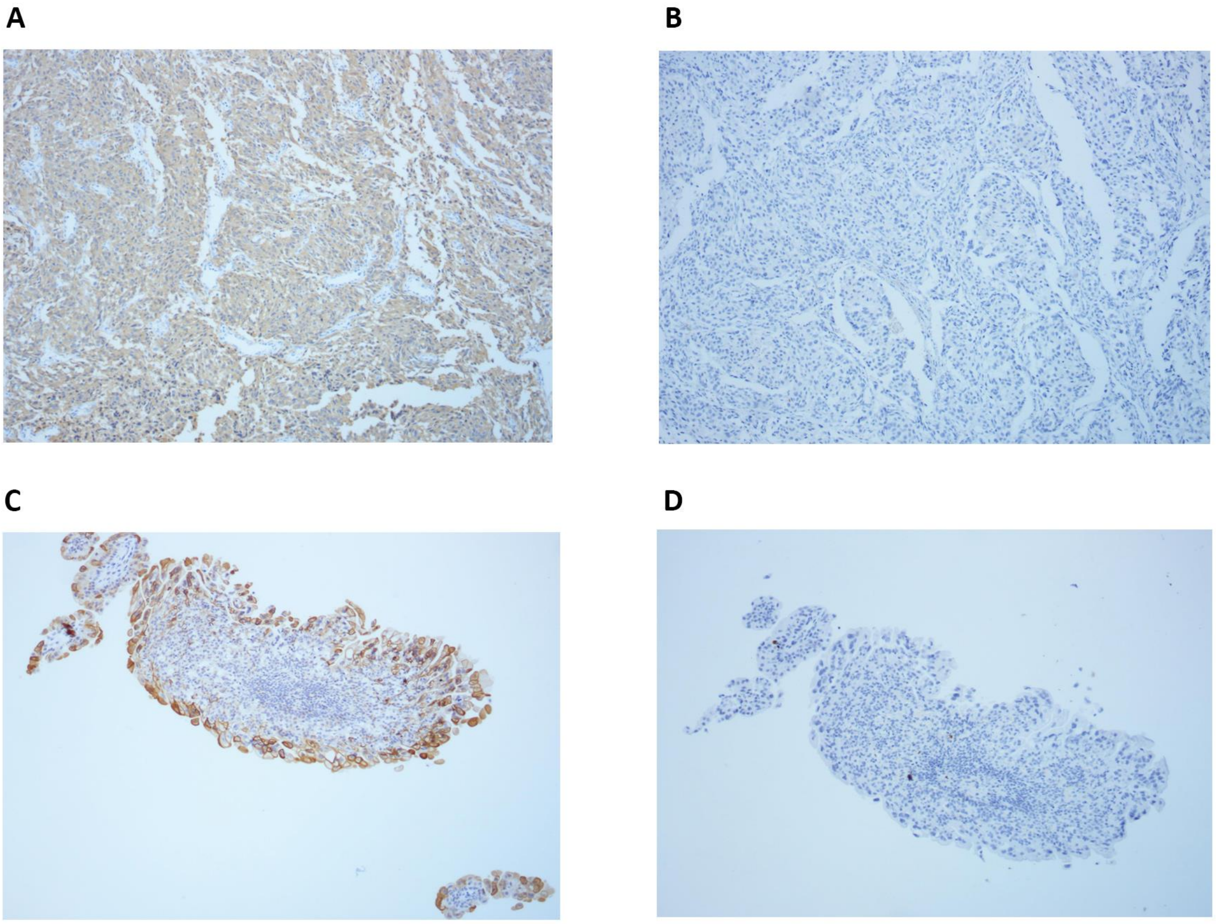

SUPPLEMENTARY FIGURE 2 The intraoperative condition and postoperative pathology results. (A, B) The results of postoperative pathology with Syn(+) (A) and S-100 (–) (B) in IHC staining of bladder pheochromocytomas. (C, D) The results of postoperative pathology with HE staining and CK20+(umbrella cells) (C) and Ki-67+(1%) (D) in IHC staining of urothelial papilloma.
