# Supplementary material for: Case Report: Octreotide plus CVD chemotherapy for the treatment of multiple metastatic paragangliomas after double resection for functional bladder paraganglioma and urothelial papilloma
Source: Front Oncol. 2023 Jan 20;12:1072361. doi: 10.3389/fonc.2022.1072361 (PMC9895770; doi:10.3389/fonc.2022.1072361)
Supplement: Supplementary Figure 3 — The results of 18F-FDG PET/CT for metastatic localizations of paragangliomas. (A–C) Multiple high uptake liver (A, B) and lung nodule (C). (D, E) High uptake localizations with osteolytic bone destruction of right parietal skull bone (D), vertebral body and ilium (E). [file Image_3.pdf]

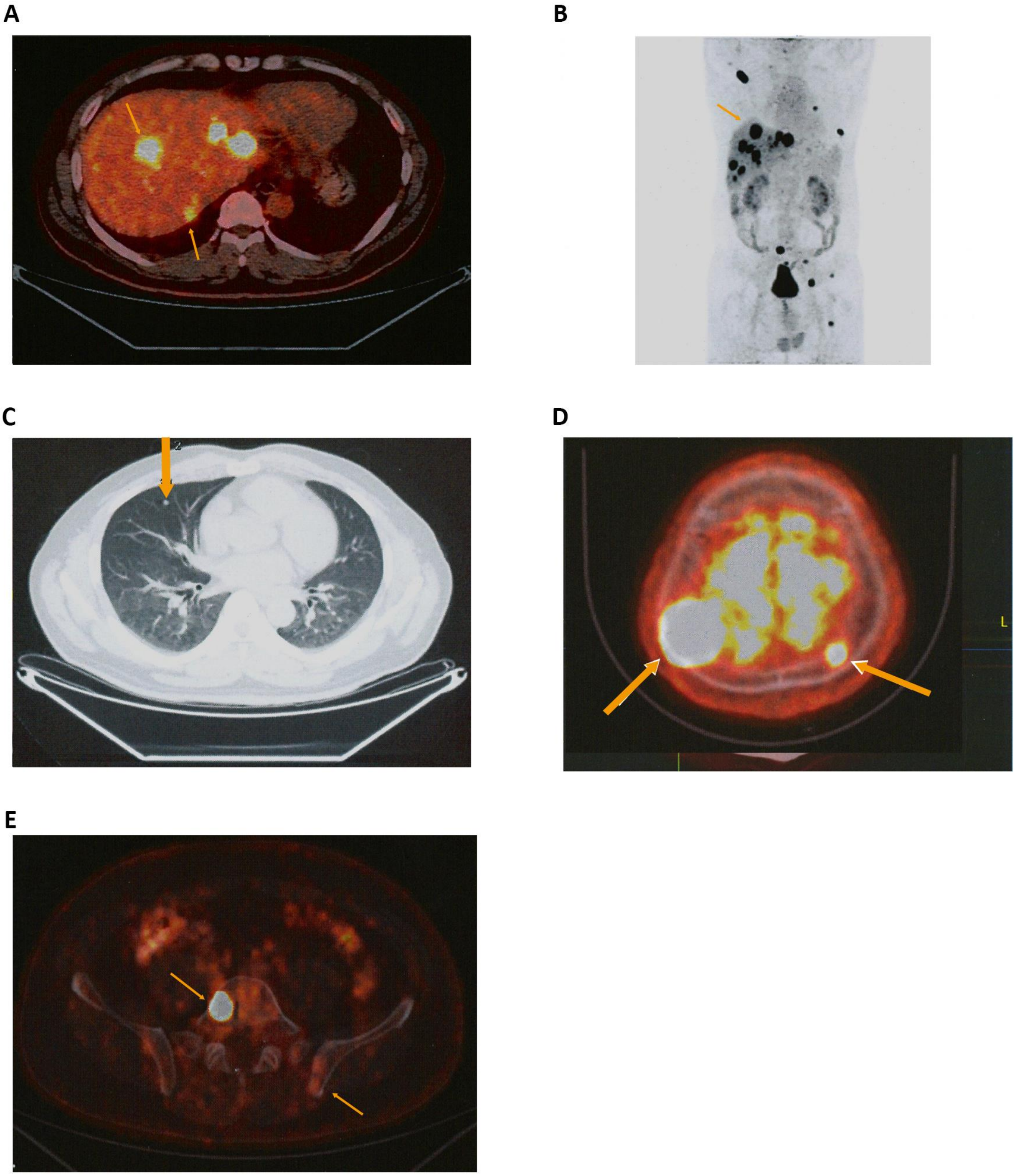

SUPPLEMENTARY FIGURE 3 The results of 18F-FDG PET/CT for metastatic localizations of paragangliomas. (A–C) Multiple high uptake liver (A, B) and lung nodule (C). (D, E) High uptake localizations with osteolytic bone destruction of right parietal skull bone (D), vertebral body and ilium (E).
